# Identification of TGF-β-related genes in cardiac hypertrophy and heart failure based on single cell RNA sequencing

**DOI:** 10.18632/aging.204901

**Published:** 2023-07-26

**Authors:** Kai Huang, Hao Wu, Xiangyang Xu, Lujia Wu, Qin Li, Lin Han

**Affiliations:** 1 Department of Cardiovascular Surgery, Changhai Hospital, Second Military Medical University, Shanghai, China

**Keywords:** single-cell RNA sequencing, TGF-β, heart failure, cardiac hypertrophy, ADAMTS2

## Abstract

Background: Heart failure (HF) remains a huge medical burden worldwide. Pathological cardiac hypertrophy is one of the most significant phenotypes of HF. Several studies have reported that the TGF-β pathway plays a double-sided role in HF. Therefore, TGF-β–related genes (TRGs) may be potential therapeutic targets for cardiac hypertrophy and HF. However, the roles of TRGs in HF at the single-cell level remain unclear.

Method: In this study, to analyze the expression pattern of TRGs during the progress of cardiac hypertrophy and HF, we used three public single-cell RNA sequencing datasets for HF (GSE161470, GSE145154, and GSE161153), one HF transcriptome data (GSE57338), and one hypertrophic cardiomyopathy transcriptome data (GSE141910). Weighted gene co-expression network analysis (WGCNA), functional enrichment analysis and machine learning algorithms were used to filter hub genes. Transverse aortic constriction mice model, CCK-8, wound healing assay, quantitative real-time PCR and western blotting were used to validate bioinformatics results.

Results: We observed that cardiac fibroblasts (CFs) and endothelial cells showed high TGF-β activity during the progress of HF. Three modules (royalblue, brown4, and darkturquoize) were identified to be significantly associated with TRGs in HF. Six hub genes (TANC2, ADAMTS2, DYNLL1, MRC2, EGR1, and OTUD1) showed anomaly trend in cardiac hypertrophy. We further validated the regulation of the TGF-β-MYC-ADAMTS2 axis on CFs activation *in vitro*.

Conclusions: This study identified six hub genes (TANC2, ADAMTS2, DYNLL1, MRC2, EGR1, and OTUD1) by integrating scRNA and transcriptome data. These six hub genes might be therapeutic targets for cardiac hypertrophy and HF.

## INTRODUCTION

As a major global health problem, heart failure (HF) exhibits progressive loss of heart function, which results in unmet metabolic and oxygen needs, and eventually leads to inevitable organ failure and death [1]. The etiology of HF varies. Hemodynamic overload-induced pathological hypertrophy is typically the beginning of HF. However, sustained cardiac hypertrophy eventually slides into the abyss of HF. However, despite updated knowledge and new technologies have been introduced into clinical practice, remedies to effectively reverse cardiac hypertrophy and HF remain lacking [2]. This highlights the significance of exploring the molecular mechanisms of cardiac hypertrophy, which may promote drug discovery for HF.

Single-cell RNA sequencing (scRNA) has been widely performed to clarify the cell heterogeneity and develop cell-type–targeted intervention during the progress of HF. The heterogeneity of cardiomyocytes from four cardiac chambers and the roles of noncardiomyocytes among normal, failed, and treated human hearts were revealed by scRNA [3]. Myocardial infarction-induced heart injury could be relieved to some extent by *in situ* myocardial injection of ACKR1^+^ endothelial cells (ECs) (3). Cardiac fibroblasts (CFs) heterogeneity during HF development in the mice model was also elucidated by scRNA, suggesting the scar-healing effect of collagen triple helix repeat containing 1-expressed CFs [4]. Furthermore, scRNA exhibited great advantages over immune cell infiltration analysis. In a transverse aortic constriction (TAC) mice model, highly expressed CD72^+^ macrophages exerted pro-inflammatory and aggravated injury effects [5], which were also confirmed in patients with dilated and ischemic cardiomyopathy.

The TGF-β signaling pathway regulates ventricular remodeling, cardiac fibrosis, and matrix metabolism during the progress of hemodynamic overload [6, 7]. Moreover, TGF-β pathway activation could exert an anti-inflammatory effect, promote myofibroblast trans-differentiation, and speed up matrix synthesis in infarcted hearts [8–10]. To the best of our knowledge, no studies focused on the TGF-β–related genes (TRGs) in HF at a single-cell level to date. Therefore, a comprehensive analysis of TRGs in HF may provide new insights into cardiac hypertrophy and HF from basic to clinical studies.

In this study, scRNA data were used to identify differentially expressed genes (DEGs) between the high and low TGF-β activity groups based on 321 TRGs. By integrating scRNA data and using multiple bioinformatics tools, including single-sample gene set enrichment analysis (ssGSEA) and weighted gene co-expression network analysis (WGCNA) in bulk sequencing data, we identified 226 genes, which were further filtered using machine learning and validated in hypertrophic cardiomyopathy dataset. A total of six hub genes (TANC2, ADAMTS2, DYNLL1, MRC2, EGR1, and OTUD1) were considered cardiac hypertrophy and HF regulators. We selected ADAMTS2 for further experimental validation and confirmed that it was downregulated in the TAC heart tissue. We further observed that ADAMTS2 overexpression could reverse the overactivation of TGF-β–induced CFs. Targeting the TGF-β-MYC-ADAMTS2 might be a novel therapy to inhibit CF overactivation and reverse cardiac hypertrophy.

## MATERIALS AND METHODS

### Quality control, batch effect correction, and cell type identification of single-cell sequencing data

The human heart scRNA datasets (GSE161470, GSE145154, and GSE161153) were downloaded from the public GEO database (https://www.ncbi.nlm.nih.gov/geo/). Four left ventricular control samples from GSE161470, three left ventricular HF samples from GSE145154, and one left ventricular sample with HF from GSE161153 were filtered out for further analysis using R (version 4.2.0) and Seurat package (version 4.1.1).

Large gene expression matrices were formed using the “merge” function. Cells with >500 genes, <5,000 genes, and <20% mitochondrial genes were retained. Gene expression lists were normalized using the “NormalizeData” function and further scaled. Subsequently, the “vst” method was used for each sample to identify 2,000 highly variable genes. According to the highly variable genes, principal component analysis was applied to identify significant principal components (PCs), which were visualized using the ElbowPlot function.

Since scRNA data were collected from three different research groups, the “Harmony” package (version 0.1.0) was used to correct the batch effect. We selected 20 PCs to execute t-distributed stochastic neighbor embedding (t-SNE) analysis. Fifteen cell clusters were classified using the “FindClusters” function with a resolution of 0.5. DEGs for each cell cluster were screened using the “FindAllMarkers” function with a threshold of 0.25. The top five DEGs for cell clusters, previously reported article [11], and CellMarker database (http://bio-bigdata.hrbmu.edu.cn/CellMarker/) were used to annotate cell types.

### TRG score

We collected eight TGF-β–related gene sets from several public databases, including GO:0007179 from AmiGO2 (http://amigo.geneontology.org/amigo), Signaling by TGF-beta Receptor Complex from Reactome (https://reactome.org/), TGF-beta signaling pathway from KEGG (https://www.genome.jp/kegg/pathway.html), PID_TGFBR_PATHWAY, BIOCARTA_TGFB_PATHWAY, WP_CANONICAL_AND_NONCANONICAL_TGFB_SIGNALING, WP_TGFBETA_RECEPTOR_SIGNALING, and WP_TGFBETA_SIGNALING_PATHWAY from GSEA (http://www.gsea-msigdb.org/gsea/msigdb/genesets.jsp). Thus, a total of 321 TRGs were collected (Supplementary Table 1).

We used the “AddModuleScore” function to score the TRG expression levels in every cell. Cells with scores greater than 75% quantile were deemed as the high TGF-β activity group, and those with scores lower than 75% quantile were the low TGF-β activity group. DEGs between those two groups were calculated using the “FindMarkers” function. Subsequently, the TGF-β scores of each cell were mapped to the t-SNE embedding and visualized using the ggplot2 package (version 3.3.6).

### Transcriptome data download and processing

The microarray data of GSE57338 (177 and 136 human left ventricular heart samples with and without HF) and RNA-seq data of GSE141910 (28 and 166 healthy human left ventricular samples with and without cardiac hypertrophy, respectively) were obtained from the GEO database. DEGs of datasets were calculated using “limma” package (version 3.52.1) in R. The threshold for DEGs was logFC > mean (abs (logFC) + 2 * SD (logFC)) and *P* < 0.05.

### ssGSEA and WGCNA

The ssGSEA was used to calculate the TGF-β score of each sample in GSE57338 based on the 321 TRGs.

In this study, WGCNA was performed to determine the gene modules most related to TGF-β scores. Briefly, following soft-thresholding power selection, the adjacency matrix was formed and turned into topological overlap. The clustering tree was plotted using the hierarchical clustering method. Genes in the expression data of GSE57338 were aligned to different modules (minModularSize = 50), and similar modules were merged using the “DynamicTreeCut” algorithm (cutHeight = 0.3). The “LabeledHeatmap” function was used to depict the relationship between different modules and TGF-β scores. The random seed was set as 123.

### Gene ontology (GO), Kyoto Encyclopedia of Genes and Genomes (KEGG), and GSEA analysis

“EnrichGO,” “EnrichKEGG,” and “gseKEGG” functions from clusterProfiler package (version 4.4.2) were applied for GO, KEGG, and GSEA, respectively. *P* < 0.05 was considered statistically significant.

### Machine learning and hub gene verification

To further ascertain the hub genes, three machine learning methods, including the least absolute shrinkage and selection operator (LASSO) regression by glmnet package (versions 4.1–4), support vector machine-recursive feature elimination (SVM-RFE) by e1071 package (versions 1.7–11), and Random Forest by randomForest package (versions 4.7–1.1) were used.

The gene expression matrix of GSE57338 and corresponding grouping information were uploaded into these three algorithms. The random seed was set as 12345. Genes that came from the intersection of three algorithms were considered hub genes and subjected to further analysis.

Receiver operator characteristic curve (ROC) analysis was applied in GSE141910 to test the discrimination ability of hub genes for hypertrophic cardiomyopathy. Genes with an area under the ROC curve (AUC) of >0.7 were considered cardiac hypertrophy and HF-related hub genes.

### Construction of transcription factors (TFs) regulatory network

**Figure 1:**
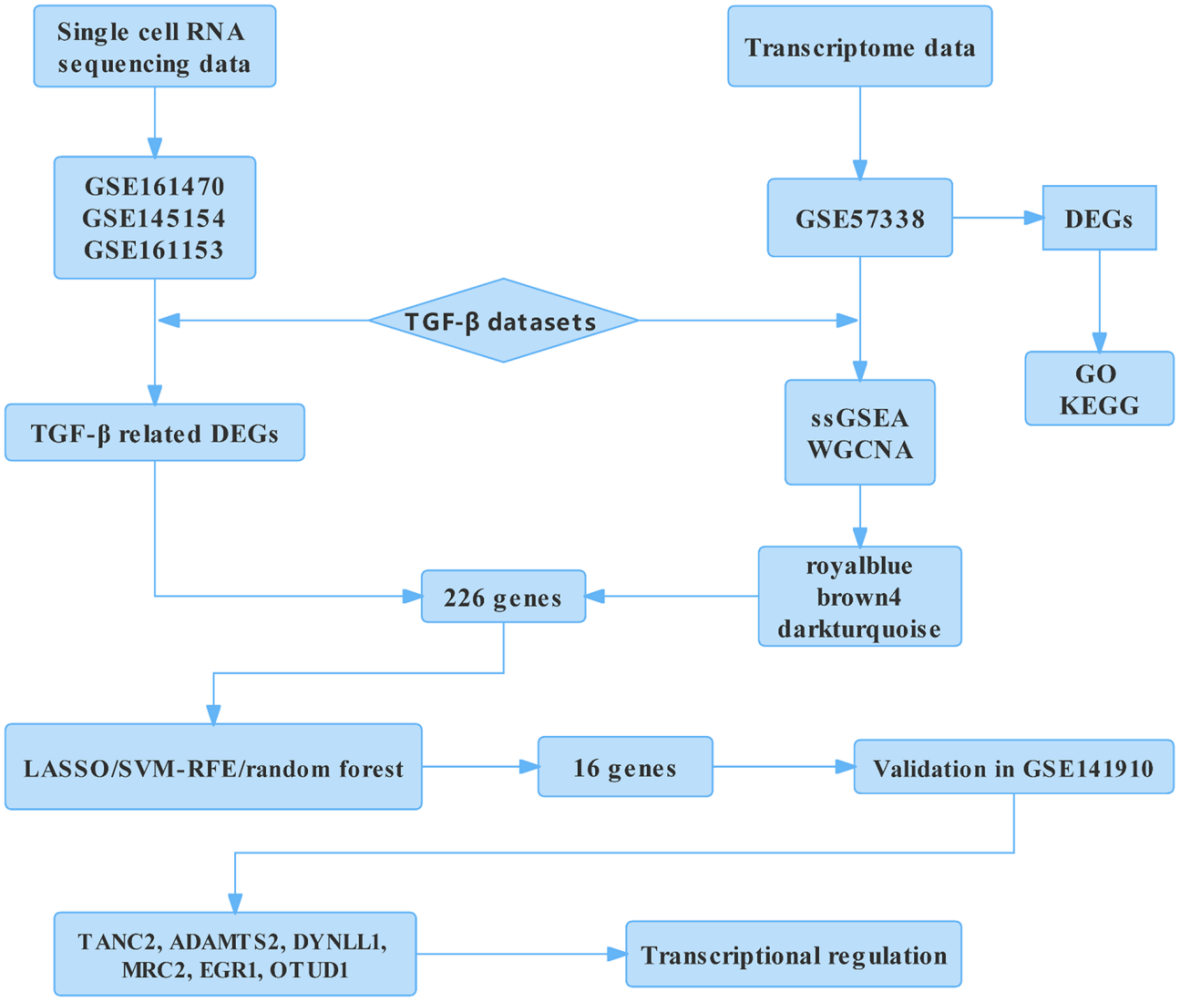
**Flow chart of the analysis.** DEGs, differentially expressed genes; GO, gene ontology annotation; KEGG, Kyoto Encyclopedia of Genes and Genomes; ssGSEA, single-sample gene Set enrichment analysis; WGCNA, weighted gene co-expression network analysis; LASSO, least absolute shrinkage and selection operator; SVM-RFE, support vector machine-recursive feature elimination; TANC2, tetratricopeptide repeat, ankyrin repeat, and coiled-coil containing 2; ADAMTS2, ADAM metallopeptidase with thrombospondin type 1 motif 2; DYNLL1, dynein light chain LC8-type 1; MRC2, mannose receptor C type 2; EGR1, early growth response 1; OTUD1, OTU deubiquitinase 1.

Differentially expressed transcription factors (DETFs) in GSE57338 were filtered out based on the TFs list downloaded from the Animal Transcription Factor Database (http://bioinfo.life.hust.edu.cn/AnimalTFDB/#!/). The “cor.test” function was used to calculate the correlation between DETFs and hub genes selected above. The thresholds were |correlation coefficient| > 0.4 and *P* < 0.05. Cytoscape software (version 3.8.2) was used to visualize the transcription regulatory network. The flow chart of this study is presented in Figure 1.

### Cell culture and treatment

CFs were isolated from neonatal rats. Briefly, heart ventricles were dissected, cut into pieces as small as possible, and digested with 0.1% Collagenase II (LS004176, Worthington, OH, USA) for 15 min at 37° C. The supernatant was collected and mixed with culture medium (high-glucose DEME with 10% FBS, 1% penicillin/streptomycin). This digestion procedure was repeated several times until tissue sediments almost disappeared. Subsequently, all the collected cell suspension was filtered through a 100-μm cell strainer and centrifuged at 1,500 rpm for 3 min. Cells were resuspended with a culture medium and placed in a cell incubator for 120 min. Newborn rat cardiomyocytes (NRCMs) were in the supernatant, whereas the remaining adherent cells were in the CFs. The cell medium was changed every other day. The passage of 2–3 CFs would be used for further experiments.

To simulate the activated condition of CFs, 10 μg/L TGF-β1 (HY-P70648, MedChemExpress, NJ, USA) was added to the cell medium. CFs were collected for the experiment at designated time points. The control group was added with the same volume of PBS.

### Cell viability and wound healing assay

CFs were seeded in 96-well culture plates with 10,000 cells per well. CCK-8 reagent (Dojindo, Japan) was added to calculate the cell viability at 490 nm absorbance using a microplate reader (BioTek, VT, USA).

Wound healing assay was used to test the migration ability of CFs in different groups. CFs were cultured in 6-well plates to approximately 80% confluence. Artificial scratches were formed using a 100-μL pipette tip. Suspended cells were washed away using PBS. Light microscope (IX70, Olympus) was used to view the cell migration distance at 24 h.

### TAC model

To induce cardiac hypertrophy according to one reported study, TAC surgery was performed [12]. Briefly, 8-week C57BL/6 mice were anesthetized with 2% isoflurane mixed with 0.5 L/min 100% O_2_. The sternum was cut to the second rib to expose the surgical field. After gently separating the thymus tissue, two loose knots were tied around the transverse aorta using 6-0 silks. The first knot was tied against a 27-G needle placed parallel to the transverse aorta, followed by the second knot and quick needle removal. The chest and skin were closed using 5-0 silks. In the sham group, the entire procedures were performed, except for aortic ligation.

### Cell transfection

CFs were transiently transfected using Myc siRNA (si-Myc), Adamts2 siRNA (si-Adamts2), siRNA negative control (si-NC), Myc plasmid (oe-Myc), Adamts2 plasmid (oe-Adamts2), empty plasmid (oe-NC) using INTERFERin (Polyplus, Illkirch, France), and jetOPTIMUS (Polyplus, Illkirch, France) according to the manufacturer’s protocols. After 8 h, the cell medium was replaced with a fresh medium. After 48 h, the cells were collected for subsequent experiments. The si-Myc sequence was 5′-AACGUUAGCUUCACCAACAUU-3′. The si-Adamts2 sequence was 5’-CCCACUGUAAAGUGGUGAAdTdT-3’. The si-NC sequence was 5′-AATTCTCCGAACGTGTCACGT-3′. si-Myc, si-Adamts2, si-NC, oe-Myc, oe-Adamts2, and oe-NC were synthesized and constructed by OBiO Technology (Shanghai, China).

### Real-time quantitative PCR (RT-qPCR)

The total RNA was isolated from CFs and heart tissues using TRIzol reagent (Thermo Fisher Scientific, MA, USA). The first-strand cDNA was synthesized from 2,000 ng of the total RNA using PrimeScript™ RT Master Mix (TaKaRa, Japan). The mRNA levels of genes were determined using real-time qPCR analysis on a LightCycler 480 II system (Roche, Switzerland) using TB Green Premix Ex Taq™ II (TaKaRa, Japan). The levels of detected mRNA were calculated using the 2^-ΔΔCt^ method.

### Western blot analysis

After treatment, cell and heart tissues were lysed with RIPA buffer (P0013B, Beyotime, China) and incubated for 30 min at 4° C. The protein solution was centrifuged at 12,000 rpm for 15 min at 4° C. BCA Protein Assay Kit (P0012S, Beyotime, China) was used to calculate the protein concentration. Then, 40 μg of protein were separated by SDS-PAGE gels and transferred to PVDF membranes (IPVH00010, Millipore, MA, USA). The membranes were then blocked with 5% defatted milk for 1 h and incubated with a primary antibody at 4° C overnight. Subsequently, the membranes were incubated with HRP-linked secondary antibody (1:5,000) at room temperature for 1.5 h. To analyze signal intensities, the chemiluminescent system (Affinity™ ECL kit, Affinity Biosciences, OH, USA) and Gel Doc XR+ Gel Documentation System (Bio-Rad, CA, USA) were used. The following were the antibodies used: anti-ADAMTS2 (A10272, ABclonal, China), anti-Collagen I (66761-1-Ig, Proteintech, IL, USA), anti-α-SMA (14395-1-AP, Proteintech, IL, USA), and anti-GAPDH (60004-1-Ig, Proteintech, IL, USA).

### Dual luciferase reporter gene assay

The three most possible binding sites between MYC and promoter of ADAMTS2 were predicted using the NCBI website (https://www.ncbi.nlm.nih.gov/gene/) and the JASPAR database (http://jaspar.genereg.net/). Subsequently, the wild-type ADAMTS2 promoter (0 to +2,000 bp) and three mutated ADAMTS2 promoters were subcloned into pGL3-basic luciferase reporter vectors to construct pGL3-WT-ADAMTS2, pGL3-MUT1-ADAMTS2 (+1,139 to +1,150 bp), pGL3-MUT2-ADAMTS2 (+607 to +618 bp), and pGL3-MUT3-ADAMTS2 (+1,919 to +1,930 bp). These four vectors were co-transfected with the MYC overexpression vector (OE-MYC) into 293 cells, respectively. The Dual-Lumi™ Luciferase Reporter Gene Assay Kit (RG089S, Beyotime, China) was used to detect the firefly luciferase activity (relative to the Renilla luciferase activity) of the target reporter gene.

### Chromatin immunoprecipitation (ChIP)

The ChIP kit (Absin, abs50034) was used to perform ChIP experiments. First, cells were crosslinked and fixed by formaldehyde, and the DNA was broken into suitable fragments by ultrasonography. Subsequently, the corresponding antibody was used to bind the specific DNA fragment. Finally, DNA fragments were purified using a DNA purification column. An antibody against MYC for ChIP was obtained from Proteintech (67447-1-Ig). The enrichment efficiency of the binding site was determined using qRT-PCR. The primes (forward: 5’-AGGTGTCCTTGGATGCTTGG-3’ and reverse: 5’-TATGCATTCTGTCCTCCCGC-3’) were designed for site 1. The distal primers (forward: 5′-CACCCAAGATGACCCGGAAA-3′ and reverse: 5′-GTGAGGACAAGTCAGCGTCA-3′) were designed as the control for site 1. The distance from the transcription start site for ADAMTS2 is 4,000 bp.

### Statistical analyses

Statistical analyses were performed using GraphPad Prism 9.0. At least three independent experiments were performed for each assay. Unpaired, two-tailed t-test was used to compare the two groups. Data from multiple groups were analyzed using one-way analysis of variance (ANOVA). To assess intergroup differences, two-way ANOVA was used. *P* < 0.05 was considered statistically significant.

### Data availability

All the datasets used in this study can be downloaded from online GEO database, including GSE161470 (https://www.ncbi.nlm.nih.gov/geo/query/acc.cgi?acc=GSE161470), GSE145154 (https://www.ncbi.nlm.nih.gov/geo/query/acc.cgi?acc=GSE145154), GSE161153 (https://www.ncbi.nlm.nih.gov/geo/query/acc.cgi?acc=GSE161153), GSE57338 (https://www.ncbi.nlm.nih.gov/geo/query/acc.cgi?acc=GSE57338), and GSE141910 (https://www.ncbi.nlm.nih.gov/geo/query/acc.cgi?acc=GSE141910). The raw data supporting the conclusions of this article will be made available by the authors, without undue reservation.

## RESULTS

### scRNA profile of human heart tissue

**Figure 2:**
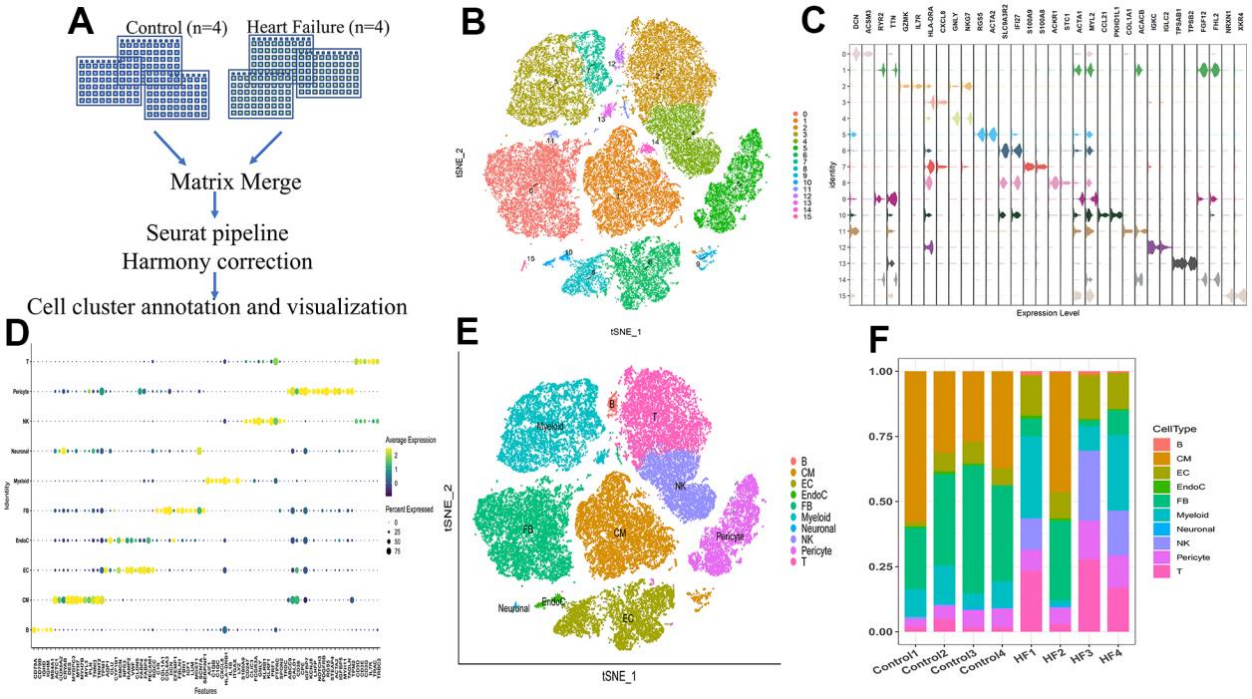
**Single-cell RNA sequencing shows the heterogeneity of the heart tissue.** (**A**) Pipeline of single-cell RNA sequencing data processing. (**B**) t-SNE plot representing the 15 clusters across 39,995 cells from four controls and four heart failure samples. (**C**) Violin plots showing the expression of marker genes for the 15 cell clusters. (**D**) Dot plot showing the expression of the top five DEGs in each cell type. (**E**) t-SNE plot representing the 10 cell clusters after annotation. B, B cells; CM, cardiac muscle cells; EC, endothelial cells; EndoC, endocardial endothelial cells; FB, fibroblasts; myeloid, myeloid cells; neuronal, neurogenic cells; NK, natural killer cells; T, T cells. (**F**) Bar plot showing the proportion of cell types in each sample.

The scRNA data of 46,180 cells from four heart tissues without failure and four HF samples were analyzed (Figure 2A). After raw data processing and filtration (Supplementary Figure 1A–1D), a total of 39,995 cells were retained. Followed by gene matrix normalization, 15 cell clusters were identified using 20 PCs (Figure 2B and Supplementary Figure 1E).

Based on marker genes (Figure 2C and Supplementary Table 2) and top five DEGs (Figure 2D and Supplementary Table 3), cells could be assigned to 10 known kinds of different cells (Figure 2E). The proportion of each cell type showed great heterogeneity among the control and HF samples (Figure 2F).

### TGF-β scores of heart cell clusters

To illuminate the expression pattern of TRGs in cell clusters, we used the “AddModuleScore” function to calculate the TGF-β score of each cell based on the collected 321 TRGs.

**Figure 3:**
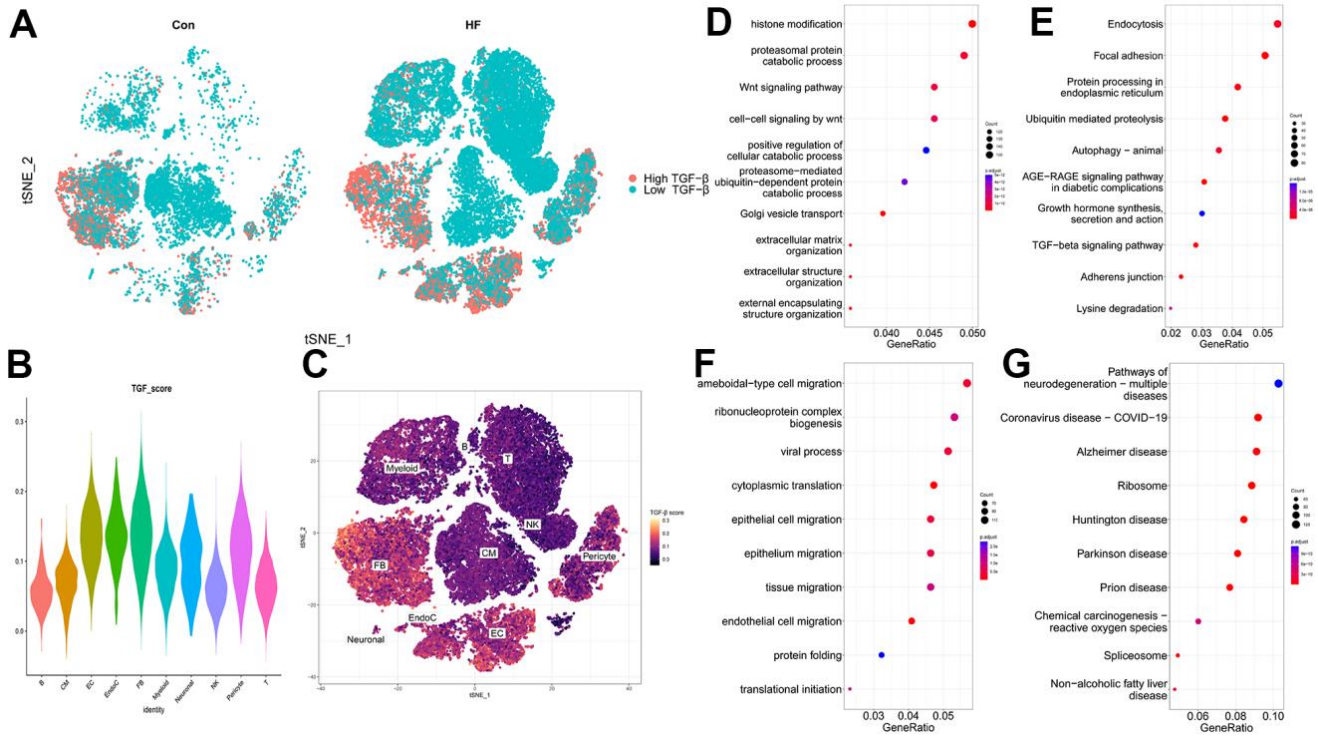
**TGF-β score heart failure cell clusters.** (**A**) t-SNE plot showing the high and low TGF-β activity in the control and heart failure samples. (**B**) TGF-β score for each cell cluster. (**C**) Heatmap showing the TGF-β activity. (**D**, **E**) GO and KEGG enrichment analyses of DEGs for CFs. (**F**, **G**) GO and KEGG enrichment analyses of DEGs for ECs.

Cells expressing more TRGs showed higher TGF-β scores. As shown in Figure 3A, more cells in the HF samples are expressing TRGs, indicating that TGF-β–related pathways are activated in the HF group. Particularly, CFs and ECs were the main target cell clusters of TRGs (Figure 3B, 3C).

GO and KEGG were used to investigate the functional characteristic of DEGs from CFs and ECs. For CFs, the expression features were mainly related to histone modification, proteasomal protein catabolic process, Wnt signaling pathway (Figure 3D), endocytosis, focal adhesion, and protein processing in the endoplasmic reticulum (Figure 3E). For ECs, amoeboid-type cell migration, ribonucleoprotein complex biogenesis, viral process (Figure 3F), pathways of neurodegeneration-multiple diseases, coronavirus disease, and Alzheimer disease (Figure 3G) were the main enrichment results.

### DEGs of HF from transcriptome data

**Figure 4:**
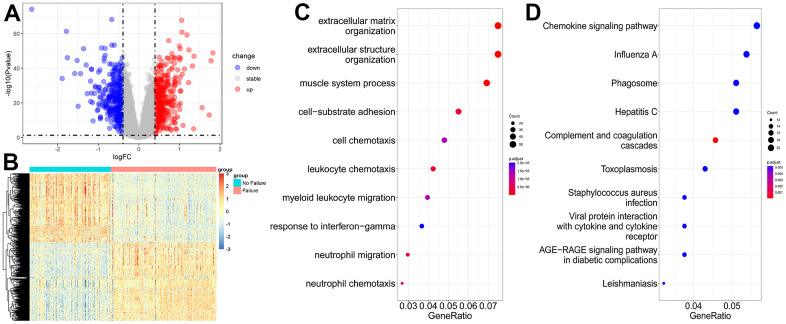
**Transcriptome data analysis for heart failure.** (**A**) Volcano plot of DEGs for no failure and heart failure samples. (**B**) Heatmap of DEGs for no failure and heart failure samples. (**C**, **D**) GO and KEGG enrichment analyses of DEGs for no failure and heart failure samples.

The transcriptome dataset GSE57338 (136 controls and 177 HF samples) was used to explore the gene expression features of HF at the tissue level. The thresholds for DEGs were |logFC| > 0.394 and *P* < 0.05. A total of 414 upregulated and 362 downregulated DEGs were selected (Supplementary Table 4). The volcano map and heatmap for these DEGs are presented in Figure 4A, 4B.

GO and pathway enrichment analyses showed that most of these DEGs were focused on extracellular matrix organization, extracellular structure organization, muscle system process (Figure 4C), chemokine signaling pathway, influenza A, and phagosome (Figure 4D).

### TGF-β activity-related gene modules in transcriptome data

**Figure 5:**
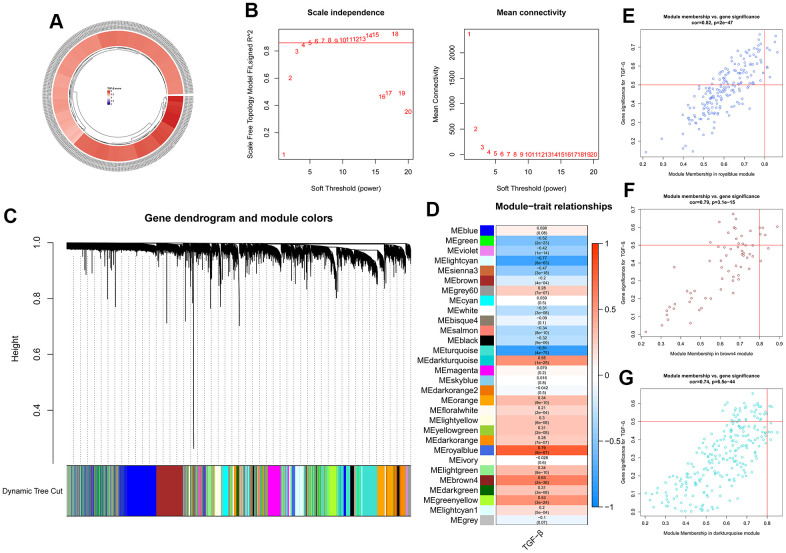
**ssGSEA and WGCNA results.** (**A**) Circular heatmap showing the TGF-β scores of 313 samples calculated using ssGSEA. (**B**) Analysis of the network topology for various soft-thresholding powers. (**C**) Clustering dendrogram of genes. (**D**) Heatmap showing the correlation between modules and TGF-β scores. (**E**–**G**) Three gene modules selected for further analysis.

ssGSEA was used to score each sample of GSE57338 based on TRGs. The TGF-β scores were shown as a circular heatmap (Figure 5A). To seek the most significant gene modules related to TRGs, gene matrix and score information were input to WGCNA. The soft-thresholding power of five was selected (Figure 5B), and all the genes could be allotted into 30 modules (Figure 5C, 5D). Among them, three gene modules were selected for further analysis, including royalblue (Figure 5E), brown4 (Figure 5F), and darkturquoize (Figure 5G), which were the top three modules most related to TGF-β scores.

**Figure 6:**
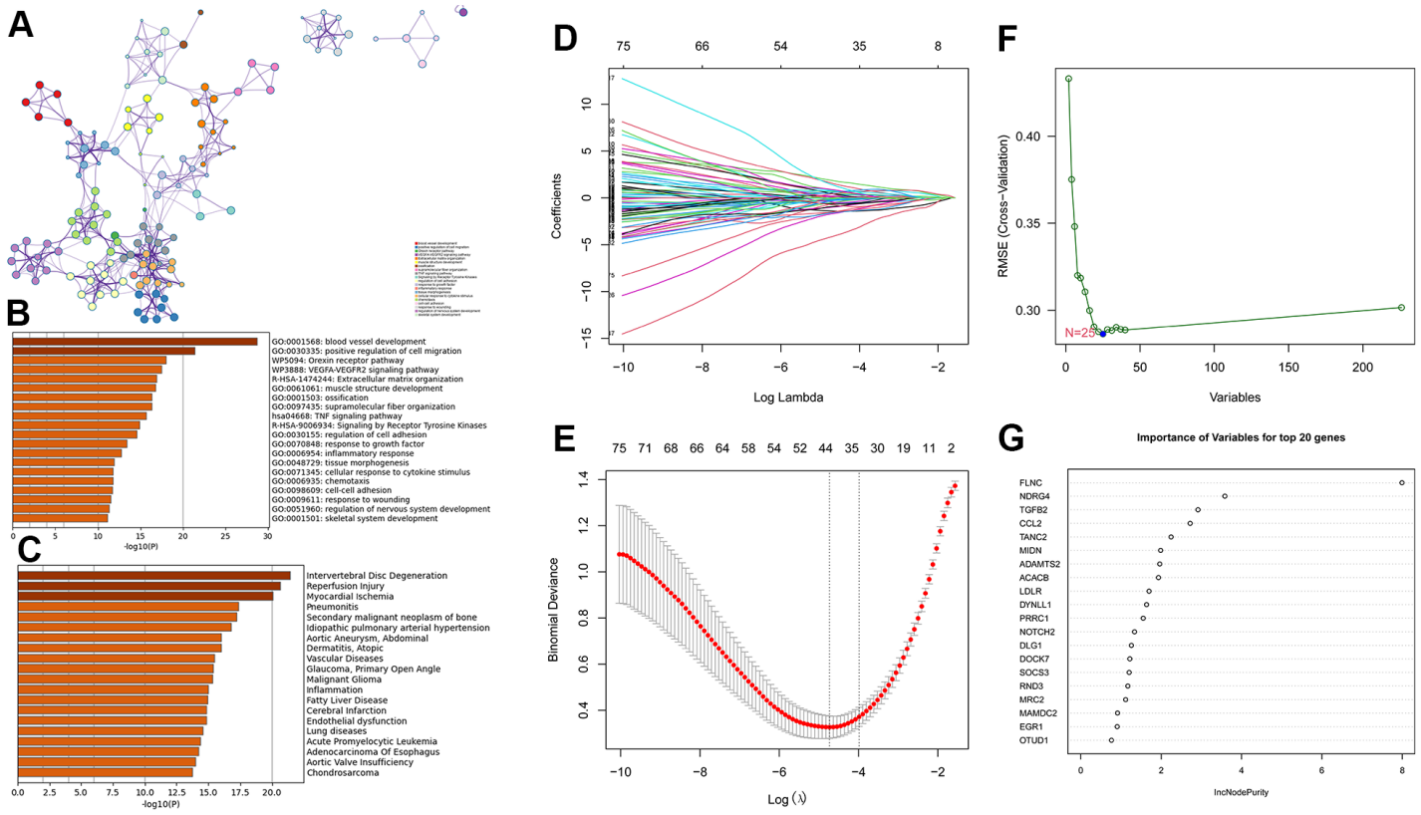
**Enrichment analysis for the three gene modules (royalblue, brown4, and darkturquoize) using Metascape and machine learning.** (**A**, **B**) The network and bar plot of enriched terms for genes in the three gene modules. (**C**) The enriched diseases for genes in the three gene modules using the DisGeNET database. (**D**, **E**) A total of 44 genes identified using the LASSO regression. (**F**) A total of 25 genes were identified using the SVM-RFE algorithm. (**G**) A total of 20 genes were identified using random forest.

A total of 502 genes in these three modules (Supplementary Table 5) were uploaded to Metascape (https://metascape.org) for enrichment analysis. As shown in Figure 6A, 6B, those genes are mostly enriched in blood vessel development, positive regulation of cell migration, orexin receptor pathway, VEGFA-VEGFR2 signaling pathway, and muscle structure development. Intervertebral disc degeneration, reperfusion injury, myocardial ischemia, pneumonitis, secondary malignant neoplasm of the bone, and idiopathic pulmonary arterial hypertension were the most related diseases of the abovementioned genes (Figure 6C).

### Hub gene verification and TF regulatory network construction

**Figure 7:**
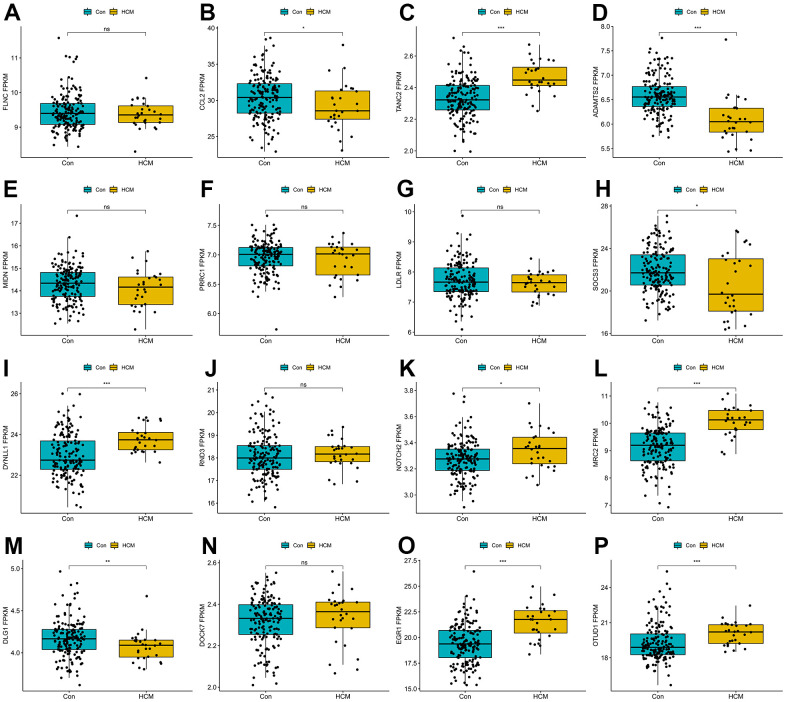
**Boxplots of the sixteen genes in hypertrophic cardiomyopathy dataset (GSE141910).** (**A**) FLNC; (**B**) CCL2; (**C**) TANC2; (**D**) ADAMTS2; (**E**) MIDN; (**F**) PRRC1; (**G**) LDLR; (**H**) SOCS3; (**I**) DYNLL1; (**J**) RND3; (**K**) NOTCH2; (**L**) MRC2; (**M**) DLG1; (**N**) DOCK7; (**O**) EGR1; (**P**) OTUD1. ns P > 0.05, * P < 0.05, ** P < 0.01, *** P < 0.001.

**Figure 8:**
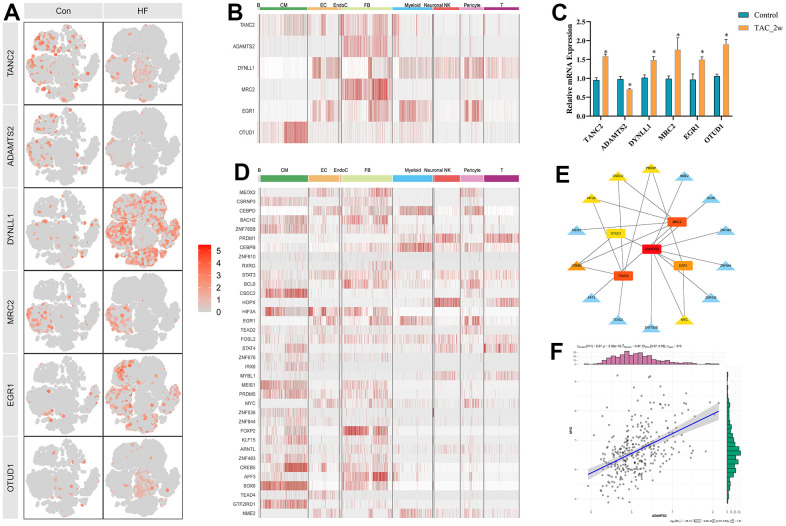
**Six hub genes and corresponding transcriptional factors.** (**A**) t-SNE plot showing the expression characteristic of the six hub genes in the control and heart failure samples. (**B**) Heatmap showing the expression of six hub genes in each cell type. (**C**) Relative mRNA expression of the six hub genes in the 2w TAC model. * P < 0.05. (**D**) Heatmap showing the expression of differentially expressed transcriptional factors in each cell type. (**E**) Transcriptional factor and hub gene regulatory network. (**F**) Correlation diagram between MYC and ADAMTS2.

To further narrow the range of candidate hub genes, DEGs derived from the low and high TGF-β activity groups in scRNA sequencing data and genes in three modules from WGCNA were taken intersection. Thus, 226 genes were filtered out (Supplementary Table 6). Subsequently, three machine learning algorithms, including LASSO (family = “binomial”, alpha=1, Figure 6D, 6E), SVM-RFE (k=10, halve.above=50, Figure 6F), and random forest (ntree = 500, Figure 6G), were used to decrease these genes to sixteen (Supplementary Table 7). To test the discriminability of the sixteen genes in cardiac hypertrophy, boxplots (Figure 7A–7P) and ROC (Supplementary Figure 2) were plotted in GSE141910. The expression of these sixteen genes were also evaluated in GSE57338 (Supplementary Figure 3). Only six genes with AUC > 0.7, including TANC2, ADAMTS2, DYNLL1, MRC2, EGR1, and OTUD1, were retained and deemed as hub genes. The expression characteristics of the six hub genes at a single-cell level are shown in Figure 8A, 8B. ADAMTS2 and MRC2 were mainly expressed in fibroblasts (FBs). DYNLL1 was expressed in all cell types, except for CMs. Myeloids, FBs, ECs, and pericytes were the target cell types of ERG1. OTUD1 was mostly expressed in CMs. FBs and CMs showed the expression of TANC2. The mRNA expression levels of these six hub genes in the TAC_2w model were consistent with bioinformatics analysis (Figure 8C). The primers used for RT-qPCR are presented in Supplementary Table 8.

To explore the transcriptional regulatory mechanisms of these hub genes, the DEGs in GSE57338 were taken intersection with the TF list acquired from the Animal Transcription Factor Database (Supplementary Table 9), and 36 differentially expressed TFs (DETFs) were picked up (Supplementary Table 10). The expression profiles of DETFs in various cell types are presented in Figure 8D. The correlation coefficient between DETFs and hub genes is shown in Supplementary Table 11. Twenty-one DETF-hub gene pairs were visualized using Cytoscape (Figure 8E). ADAMTS2 was at the center of the regulatory network. Interestingly, MYC, the predicted transcription regulator of ADAMTS2, is also the downstream molecule of the TGF-β signaling pathway. The expression relationship of MYC-ADAMTS2 and other TF-hub gene pairs in HF, respectively, are presented in Figure 8F and Supplementary Figure 4A–4T. Therefore, we speculated that the TGF-β-MYC-ADAMTS2 axis might play a role in the development of cardiac hypertrophy and HF, which needs experimental validation.

### ADAMTS2 overexpression reverses the effect of TGF-β on CFs

**Figure 9:**
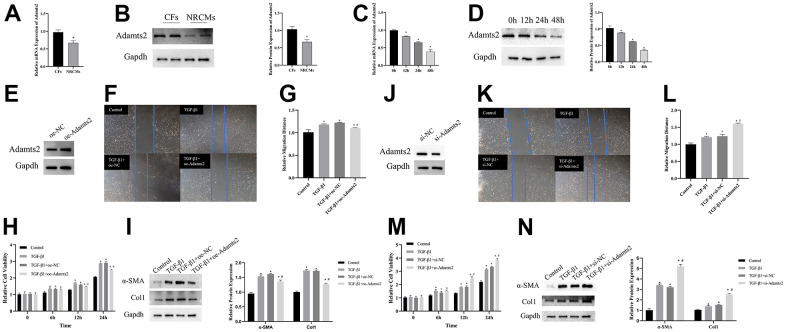
**Upregulation of ADAMTS2 alleviates the effect of TGF-β1 on cardiac fibroblasts.** (**A**) The mRNA expression of Adamts2 in CFs and NRCMs was determined by RT-qPCR. * P < 0.05. (**B**) The protein expression of Adamts2 in CFs and NRCMs was determined western blot. * P < 0.05. (**C**) The mRNA expression of Adamts2 in TGF-β1 treated CFs at different time points. * P < 0.05 vs. the 0h group. (**D**) The protein expression of Adamts2 in TGF-β1 treated CFs at different time points. * P < 0.05 vs. the 0h group. (**E**) The overexpression of Adamts2 in CFs was determined by western blot. (**F**) The migration ability of CFs was showed by wound healing assay. (**G**) The relative migration distance of Adamts2 overexpressed CFs. (**H**) The cell viability was calculated by CCK-8. * P < 0.05 vs. the Control group. # P < 0.05 vs. the TGF-β group. (**I**) The expression of α-SMA and Col1 were determined by western blot. * P < 0.05 vs. the Control group. # P < 0.05 vs. the TGF-β group. (**J**) The downregulation of Adamts2 in CFs was determined by western blot. (**K**) The migration ability of CFs was showed by wound healing assay. (**L**) The relative migration distance of Adamts2 downregulated CFs. (**M**) The cell viability was calculated by CCK-8. * P < 0.05 vs. the Control group. # P < 0.05 vs. the TGF-β group. (**N**) The expression of α-SMA and Col1 were determined by western blot. * P < 0.05 vs. the Control group. # P < 0.05 vs. the TGF-β group.

The expression of Adamts2 was evaluated in CFs and NRCMs. RT-qPCR and western blot revealed that the Adamts2 showed higher expression in CFs than that in NRCMs (Figure 9A, 9B).

In HF, TGF-β1 not only promotes cardiac fibrosis but also activates the counterregulatory pathway of TGF-β activity [13]. Therefore, following TGF-β1 treatment (0, 12, 24, and 48 h), CFs were harvested, and the expression level of Adamts2 was detected using RT-qPCR and western blot. As shown in Figure 9C, 9D, the expression levels of Adamts2 are gradually decreased at the mRNA and protein levels, which is consistent with the results in the mice TAC model.

The role of Adamts2 in the responses to TGF-β1 stimulation *in vitro* was also explored in CFs. CFs were transfected with oe-NC and oe-Adamts2 (Figure 9E), with CFs without TGF-β1 stimulation as a control. The evaluation of cell migration (Figure 9F, 9G), viability (Figure 9H), and Col1 and α-SMA expression (Figure 9I) showed that upregulated Adamts2 mitigated the effects of TGF-β1 on CFs. In the TGF-β1–induced CFs transfected with si-Adamts2 (Figure 9J), the cell migration (Figure 9K, 9L), cell viability (Figure 9M), and Col1 and α-SMA expression (Figure 9N) were substantially enhanced. The aforementioned results indicated that Adamts2 overexpression reverses the effect of TGF-β on CFs by suppressing collagen production and overactivation of CFs.

### MYC promotes transcriptional regulation of ADAMTS2

**Figure 10:**
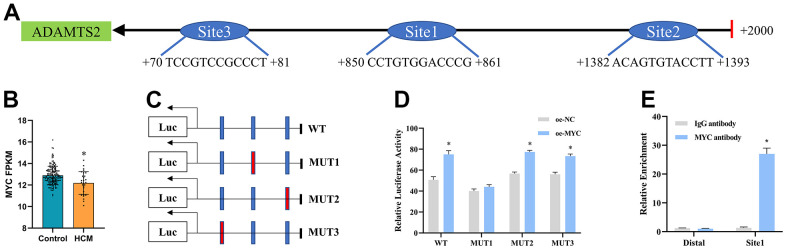
**MYC transcriptionally regulates ADAMTS2.** (**A**) Three predicted binding sites of the MYC protein in the ADAMTS2 promoter region are shown. (**B**) Boxplot showing the expression of MYC mRNA in GSE141910. * P < 0.05. (**C**, **D**) Co-transfection of the mutant ADAMTS2 promoter recombinant vector and the MYC expression vector in 293 cells is verified using dual luciferase reporter gene assays. * P < 0.05 versus the oe-NC group. (**E**) Binding of MYC to the ADAMTS2 promoters is tested using ChIP assays. * P < 0.05 versus the IgG antibody group.

We first noted that patients with hypertrophic cardiomyopathy had lower MYC levels than the control group in GSE141910 (Figure 10B). Subsequently, the three possible binding sites between MYC and the promoter of ADAMTS2 were predicted by NCBI and the JASPAR database (Figure 10A and Supplementary Figure 5). A series of recombinant luciferase reporter vectors (wild-type or mutated promoters of ADAMTS2) were constructed (Figure 10C). Results from dual luciferase reporter assay revealed that site 1 (CCTGTGGACCCG) in the promoter region of ADAMTS2 was the binding site between MYC and ADAMTS2 (Figure 10D). The binding ability of MYC to the ADAMTS2 promoter region at site 1 was further validated by ChIP in TGF-β1–induced CFs (Figure 10E). These results demonstrated that ADAMTS2 was transcriptionally regulated by MYC in CFs.

### TGF-β-MYC-ADAMTS2 axis in CFs

**Figure 11:**
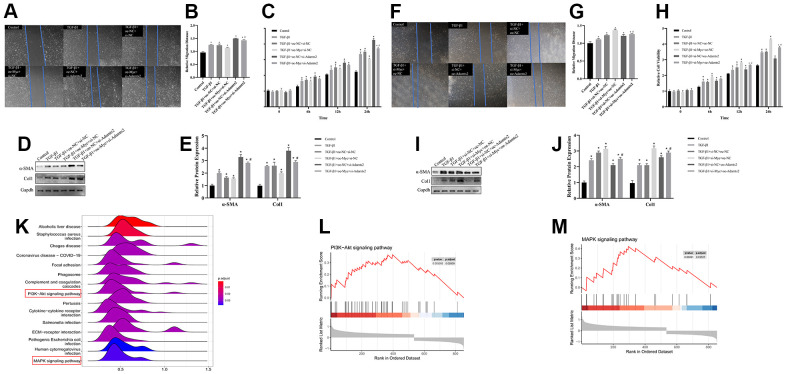
**The regulatory effect of TGF-β-MYC-ADAMTS2 axis on CFs.** (**A**) The migration ability of CFs was showed by wound healing assay. (**B**) The relative migration distance of CFs. * P < 0.05 vs. the Control group. # P < 0.05 vs. the TGF-β1+oe-NC+si-Adamts2 group. (**C**) The cell viability was calculated by CCK-8. * P < 0.05 vs. the Control group. # P < 0.05 vs. the TGF-β1+oe-NC+si-Adamts2 group. (**D**) The expression of α-SMA and Col1 was determined by western blot. (**E**) The relative protein expression of α-SMA and Col. * P < 0.05 vs. the Control group. # P < 0.05 vs. the TGF-β1+oe-NC+si-Adamts2 group. (**F**) The migration ability of CFs was showed by wound healing assay. (**G**) The relative migration distance of CFs. * P < 0.05 vs. the Control group. # P < 0.05 vs. the TGF-β1+si-NC+oe-Adamts2 group. (**H**) The cell viability was calculated by CCK-8. * P < 0.05 vs. the Control group. # P < 0.05 vs. the TGF-β1+si-NC+oe-Adamts2 group. (**I**) The expression of α-SMA and Col1 was determined by western blot. * P < 0.05 vs. the Control group. # P < 0.05 vs. the TGF-β1+si-NC+oe-Adamts2 group. (**J**) The relative protein expression of α-SMA and Col. * P < 0.05 vs. the Control group. # P < 0.05 vs. the TGF-β1+si-NC+oe-Adamts2 group. (**K**) The ridgeplot for the GESA results of ADAMTS2. (**L**) The PI3K-Akt signaling pathway for ADAMTS2. (**M**) The MAPK signaling pathway for ADAMTS2.

TGF-β1–treated CFs were transfected with oe-Myc or si-Adamts2 to analyze the regulatory effect of the TGF-β-MYC-ADAMTS2 axis on CFs. The cell viability, migration, and Col1 and α-SMA expression were reduced by oe-Myc. However, the effects of the TGF-β1 treatment on cell viability, migration, and relevant gene expression were elevated by the transfection of si-Adamts2 compared with the control cells. Furthermore, co-transfection of the oe-Myc and si-Adamts2 following TGF-β1 treatment reduced the inhibitory effect of oe-Myc on cell migration (Figure 11A, 11B), cell viability (Figure 11C), and Col1 and α-SMA expression (Figure 11D, 11E).

Subsequently, TGF-β1–treated CFs were co-transfected with si-Myc and/or oe-Adamts2. We observed that Myc knockdown in TGF-β1–treated CFs improved cell migration, viability, and Col1 and α-SMA expression, which were restored following Adamts2 overexpression (Figure 11F–11J). The uncut membranes of western blot were showed in Supplementary Figure 6. Moreover, we performed single-gene GSEA for ADAMTS2 (Figure 11K) and showed that the PI3K-Akt (Figure 11L) and MAPK (Figure 11M) pathways might play a role in the regulatory effect of ADAMTS2 on CFs. Therefore, we concluded the regulatory effect of TGF-β-MYC-ADAMTS2 on CFs.

## DISCUSSION

Several studies have reported that TRGs are essential for the progression of cardiovascular disease [7]. However, few studies have highlighted on TRGs and attempted to clarify their role in cardiac hypertrophy and HF. To the best of our knowledge, our study is the first to identify TRGs suitable for further studies by integrating scRNA and bulk transcriptome data.

In the present study, we first distinguished DEGs between the high and low TGF-β activity groups at a single-cell level. At the transcriptome data level, WGCNA was performed to identify gene modules most related to the TGF-β activity. After taking intersection, machine learning, ROC analysis, and transcriptional regulation prediction, we identified six hub genes (TANC2, ADAMTS2, DYNLL1, MRC2, EGR1, and OTUD1) and their possible TFs. Indeed, some of these hub genes have been shown to be related to HF. EGR1 transcriptionally regulated the expression of mTORC1 [13] and Kir2.1 [14] in myocardial ischemia/reperfusion (I/R) injury. On the basis of solid bioinformatics analysis, we speculated that these six genes might be potential therapeutic targets for cardiac hypertrophy and HF.

TANC2 is predicted to be involved in dense core granule cytoskeletal transport, dendritic spine development, and morphogenesis. As a scaffolding protein for neurodevelopment, TANC2 could directly interact with mTOR and inhibit its activity to affect the neuron system [15]. In patients with multiple sclerosis, TANC2 could be engaged in inflammatory and neural repair pathways [16]. Meanwhile, the genetic polymorphisms of TANC2 rs2429427 and rs1029765 were reported to be associated with calcium channel blocker responses in patients with hypertension [17]. TANC2-derived rno_circRNA_002774 was downregulated in Cyclosporin A-induced cardiotoxicity [18], which may be a novel therapeutic target in autoimmune diseases and allotransplantation.

ADAMTS2 is emerging as key participant in the pathogenesis of vascular diseases [19]. Lee, C. W et al. reported that ADAMTS2 was mostly expressed in human coronary atherosclerotic plaques, particularly in ECs and macrophages [19]. Rau, C. D et al. used a newly developed co-expression network tool to identify gene modules mostly related to HF traits and reported that Adamts2 could regulate the size of cardiomyocytes induced by β-adrenergic [20]. Adamts2 knockdown alleviated the expression of hypertrophy-related genes, including Nppa and Nppb [21]. In another study, ADAMTS2 was observed to be upregulated in human failing hearts and hypertrophic murine hearts [22]. ADAMTS2 overexpression both *in vivo* and *in vitro* reversed the prohypertrophic effect of Ang II through the PI3K-AKT signaling pathway [22]. In this study, we noted that ADAMTS2 was almost only expressed in CFs and validated that MYC transcriptionally upregulated ADAMTS2. On the basis of the bioinformatics analysis and experimental results, we believed that the TGF-β-MYC-ADAMTS2 axis might be a potential therapeutic target for cardiac hypertrophy and HF.

In recent years, DYNLL1 has been deemed as an inhibitor of neuronal nitric oxide synthase and may regulate numerous biologic processes [23–26]. Yuan, J et al. reported that once dissociated with COX4IL, DYNLL1 could increase the release of mitochondrial reactive oxygen species [27]. Furthermore, DYNLL1 is involved in TGF-β–related pathways. Merino-Gracia, J et al. proposed that DYNLL1 might function as a dimerization clamp for activin receptor type IIB (ActRIIB), a type II TGF-β superfamily receptor [28]. Luciferase reporter assay revealed that DYNLT1 binding to ActRIIB resulted in the TGF-β signaling activity inhibition [28]. By applying scRNA sequencing, Gladka, M. M et al. reported that DYNLL1 was one of the responsive factors of ZEB2, which may promote cardiac contractility and scar healing following myocardial infarction [29]. In another bioinformatics study, DYNLL1 was also upregulated in dilated cardiomyopathy [30].

The protein encoded by MRC2 takes part in extracellular matrix remodeling by collagen ligand degradation. Quite a few studies have attempted to clarify the regulatory role of MRC2 in various cancers [31, 32]. Higher MRC2 and TGF-β1 expressions were independent prognosis factors for intrahepatic metastases, and MRC2 knockdown repressed the TGF-β–induced cell migration and invasion [31]. Mrc2-deficient mice showed exacerbated renal fibrosis and renal parenchymal damage following unilateral ureteral obstruction [33]. Lanlan Li et al. reported that MRC2 knockdown inhibited mouse mesangial cell proliferation and induced cell apoptosis in the model of diabetic nephropathy [34].

Chen, S. J et al. noted that EGR1 could be induced by SMAD3, and TGF-β stimulation increased its protein and mRNA levels in human skin FBs [35]. EGR1 has been reported to regulate I/R injury through various signaling pathways [36–40]. Particularly, Fan, K et al. observed that the EGR1/miR-15a-5p/GPX4 axis increased ferroptosis in acute myocardial infarction, which aggravated myocardial cell hypoxia injury [41]. Huang, C et al. reported that EGR1 overexpression enhanced neutrophil recruitment and aggravated the ensuing I/R injury by activating the TLR4/TRIF signaling pathway [42]. Pan, J et al. noted that EGR1 downregulation alleviated cardiac injury caused by acute myocardial infarction in a TLR4/NFκB signal-dependent manner [43]. Furthermore, EGR1 regulates various cardiovascular diseases, including pulmonary hypertension [44], atherosclerosis [45–47], and cardiac hypertrophy [48–50].

OTUD1 is a deubiquitinating enzyme that is not well studied and mainly limited in immune and cancer fields [51–54]. Zhang, Z et al. reported that metastasis-repressing factor OTUD1 deubiquitinated SMAD7 at Lysine 220 and prevented its degradation [55]. In the field of cardiovascular research, Xie, J et al. reported that OTUD1 might be the target of sevoflurane-induced cardio-protection [56]; Quttainah, M et al. reported the upregulation of OTUD1 in a hypertrophic myocardium [57], which was consistent with the results of this study.

Taken together, the data presented herein strongly indicate that TRGs are involved in cardiac hypertrophy and HF. scRNA analysis was used to identify specific target cell types of hub genes under pathological conditions, which provide convenience for further research. Possible therapeutic strategies designed to regulate TGF-β pathways based on the six hub genes may provide clinical responses to reverse cardiac hypertrophy and alleviate HF.

This study had some limitations. First, this study did not distinguish different etiologies (myocardial infarction, cardiomyopathy, hemodynamic overload, and inflammation) or classification (HFrEF, HFpEF, and HFmrEF) for HF. Further investigation must be performed if appropriate public datasets will be available in the future. Second, *in vivo* experimental validation and human primary cells should be used to strengthen the results. Lastly, it could have been better if the protein expression and molecular mechanisms for the other five hub genes were verified.

## CONCLUSIONS

This study identified six hub genes (TANC2, ADAMTS2, DYNLL1, MRC2, EGR1, and OTUD1) by integrating scRNA and transcriptome data. The regulatory effect of the TGF-β-MYC-ADAMTS2 axis on CF activation was also validated *in vitro*. These six hub genes might be therapeutic targets for cardiac hypertrophy and HF. To explore the related molecular mechanisms, further experimental validation must be performed.

## Supplementary Material

Supplementary Figures

Supplementary Table 1

Supplementary Tables 2 and 3

Supplementary Table 4

Supplementary Table 5

Supplementary Table 6

Supplementary Tables 7 and 8

Supplementary Table 9

Supplementary Tables 10 and 11
