# Supplementary material for: Identification of TGF-β-related genes in cardiac hypertrophy and heart failure based on single cell RNA sequencing
Source: Aging (Albany NY). 2023 Jul 26;15(14):7187–218. doi: 10.18632/aging.204901 (PMC10415570; doi:10.18632/aging.204901)
Supplement: Supplementary Figures [file aging-15-204901-s001.pdf]

SUPPLEMENTARY FIGURES

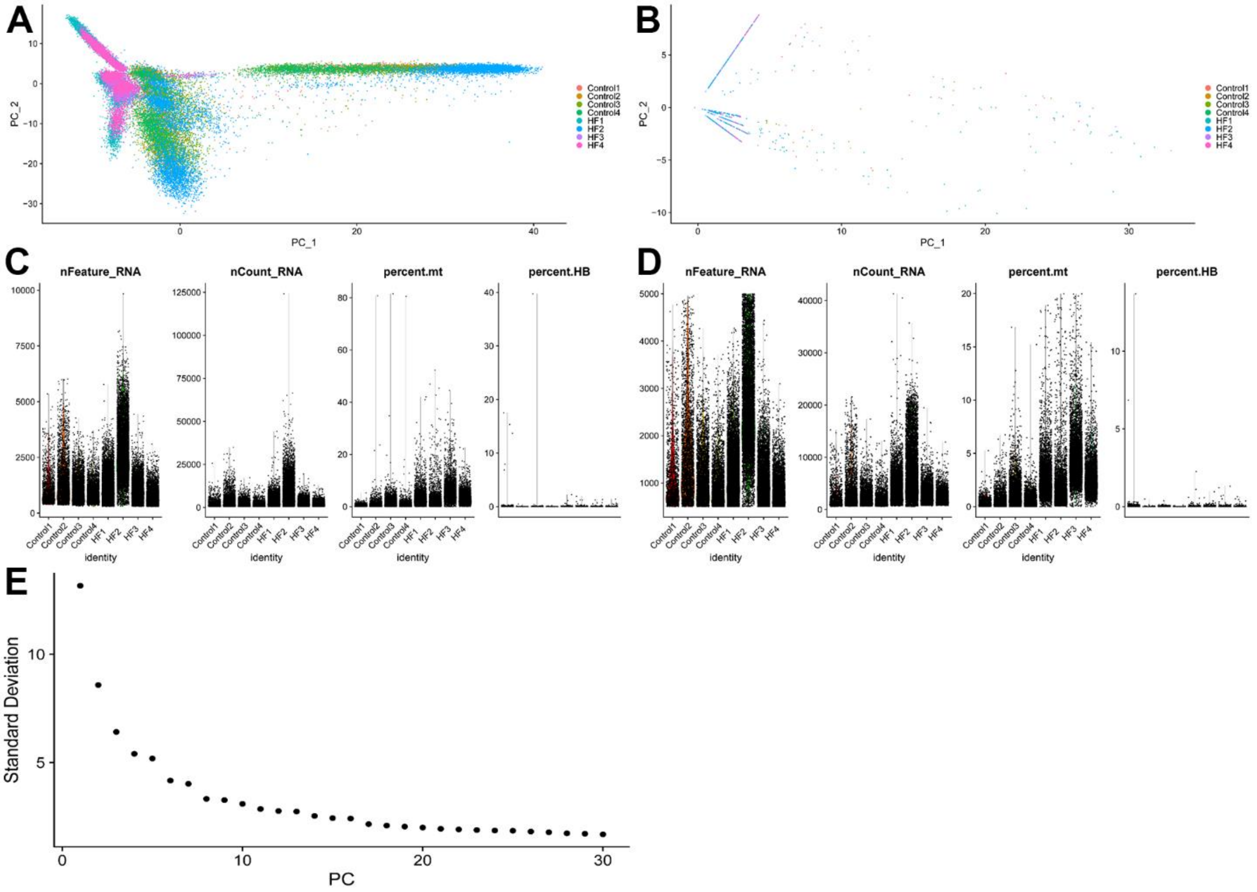

Supplementary Figure 1. Data processing and filtration of scRNA data. (**A**) Before the batch correction of 8 single cell RNA sequencing samples. (**B**) After the batch correction of 8 single cell RNA sequencing samples. (**C**) The nFeature, nCount, percent.mt and percent.HB of 8 single cell RNA sequencing samples before batch correction. (**D**) The nFeature, nCount, percent.mt and percent.HB of 8 single cell RNA sequencing samples after batch correction. (**E**) The PC selection based on Standard Deviation.

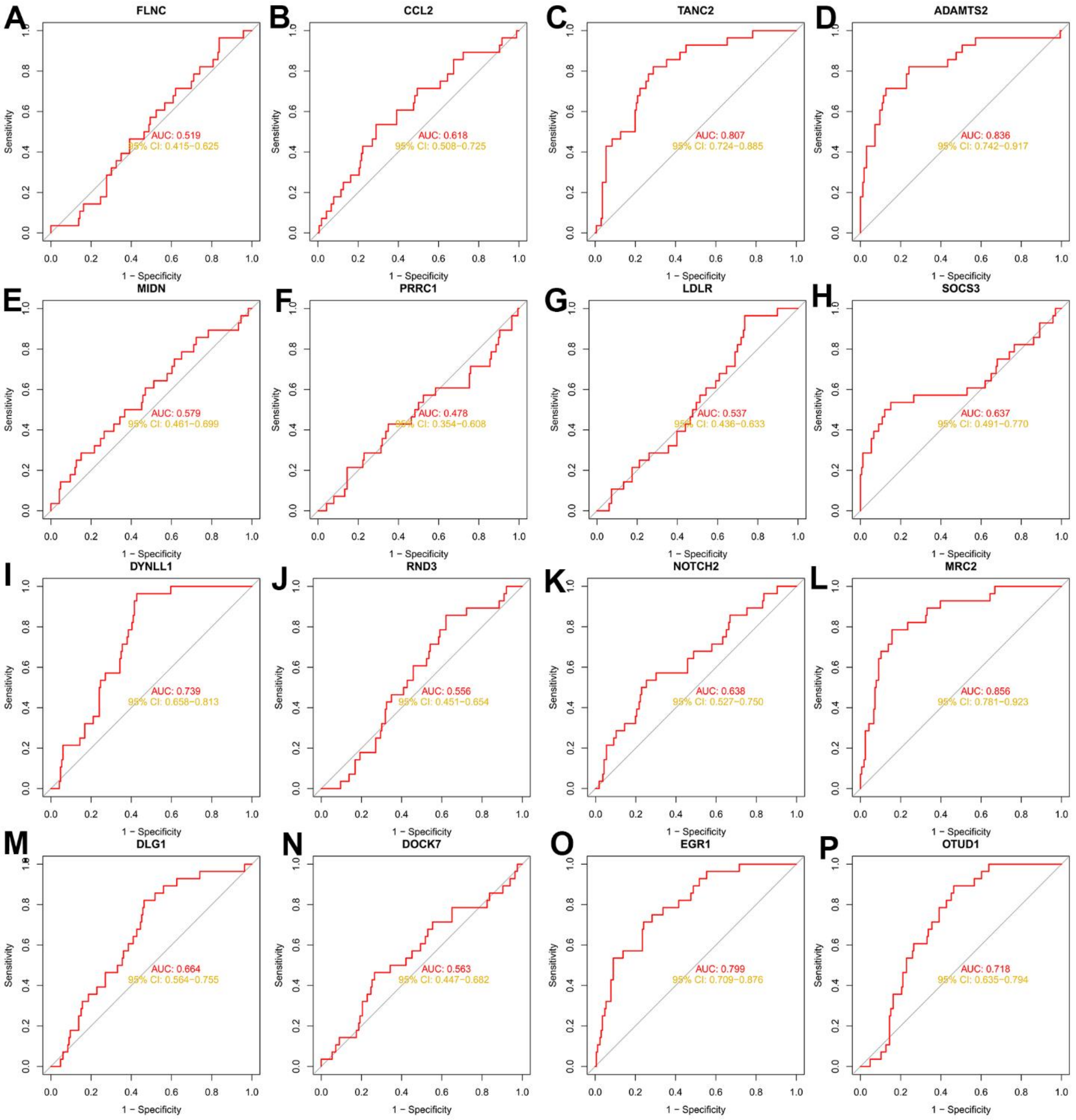

Supplementary Figure 2. ROC of 16 candidate hub genes in GSE141910. (**A**) ROC analysis of FLNC. (**B**) ROC analysis of CCL2. (**C**) ROC analysis of TANC2. (**D**) ROC analysis of ADAMTS2. (**E**) ROC analysis of MIDN. (**F**) ROC analysis of PRRC1. (**G**) ROC analysis of LDLR. (**H**) ROC analysis of SOCS3. (**I**) ROC analysis of DYNLL1. (**J**) ROC analysis of RND3. (**K**) ROC analysis of NOTCH2. (**L**) ROC analysis of MRC2. (**M**) ROC analysis of DLG1. (**N**) ROC analysis of DOCK7. (**O**) ROC analysis of EGR1. (**P**) ROC analysis of OTUD1.

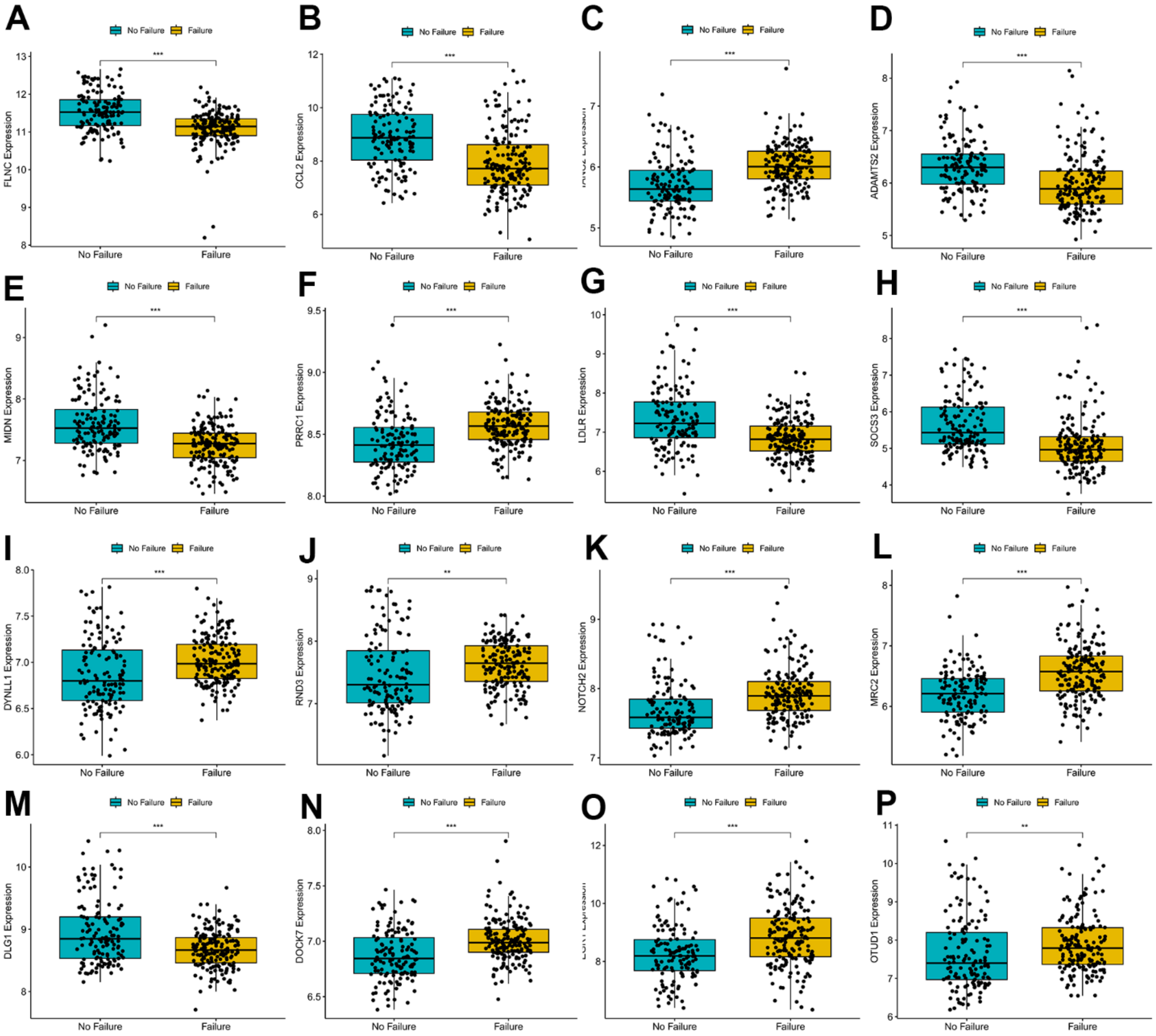

Supplementary Figure 3. Boxplots of 16 candidate hub genes in GSE57338. (**A**) Boxplot of FLNC. (**B**) Boxplot of CCL2. (**C**) Boxplot of TANC2. (**D**) Boxplot of ADAMTS2. (**E**) Boxplot of MIDN. (**F**) Boxplot of PRRC1. (**G**) Boxplot of LDLR. (**H**) Boxplot of SOCS3. (**I**) Boxplot of DYNLL1. (**J**) Boxplot of RND3. (**K**) Boxplot of NOTCH2. (**L**) Boxplot of MRC2. (**M**) Boxplot of DLG1. (**N**) Boxplot of DOCK7. (**O**) Boxplot of EGR1. (**P**) Boxplot of OTUD1.

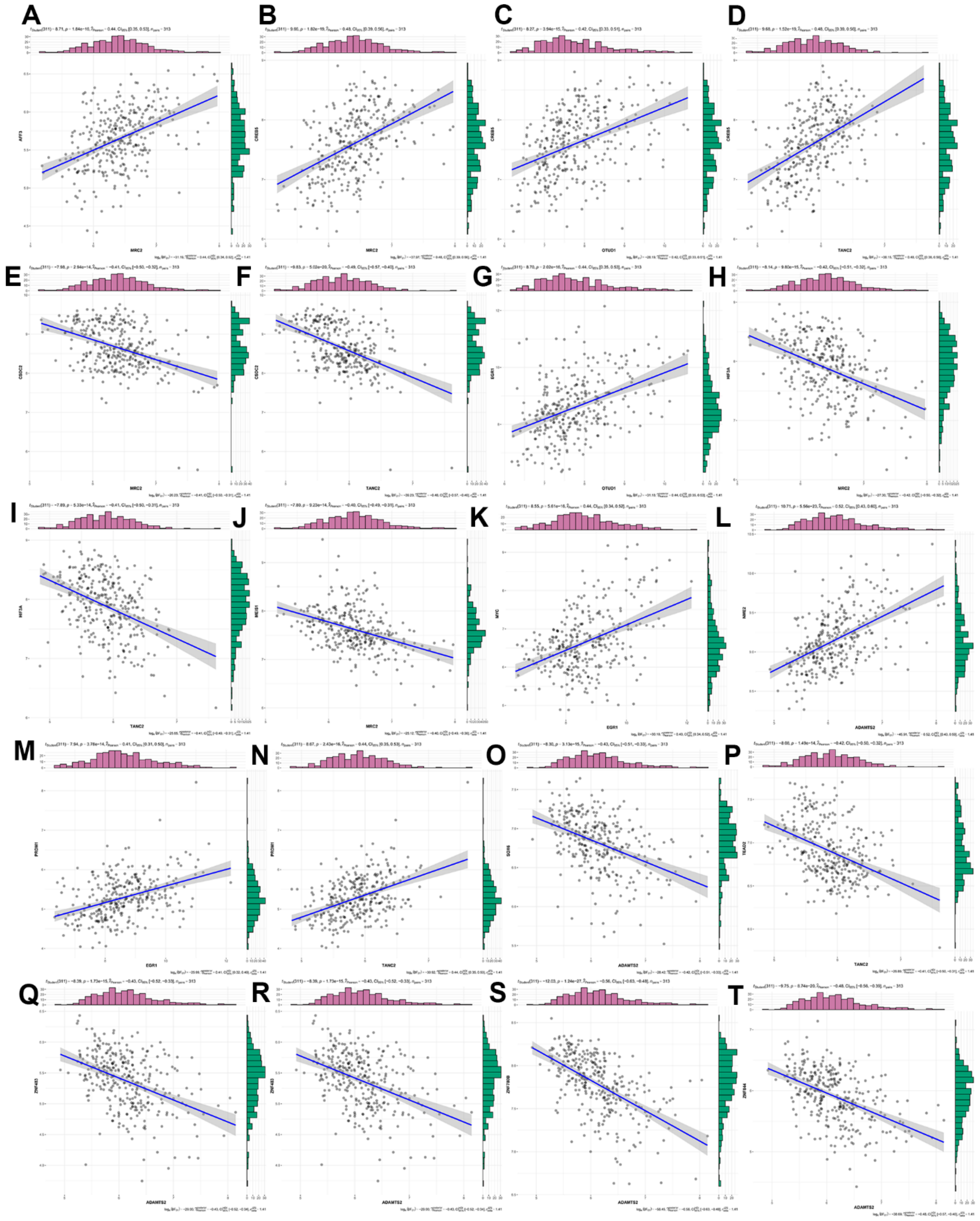

Supplementary Figure 4. Correlation diagram between DETFs and hub genes. (**A**) Correlation plot of AFF3 and MRC2. (**B**) Correlation plot of CREB5 and MRC2. (**C**) Correlation plot of CREB5 and OTUD1. (**D**) Correlation plot of CREB5 and TANC2. (**E**) Correlation plot of CSDC2 and MRC2. (**F**) Correlation plot of EGR1 and OTUD1. (**G**) Correlation plot of HIF3A and MRC2. (**H**) Correlation plot of HIF3A and TANC2. (**I**) Correlation plot of MEIS1 and MRC2. (**J**) Correlation plot of MYC and ADAMTS2. (**K**) Correlation plot of MYC and EGR1. (**L**) Correlation plot of NME2 and ADAMTS2. (**M**) Correlation plot of PRDM1 and EGR1. (**N**) Correlation plot of PRDM1 and TANC2. (**O**) Correlation plot of SOX6 and ADAMTS2. (**P**) Correlation plot of TEAD2 and TANC2. (**Q**) Correlation plot of ZNF483 and ADAMTS2. (**R**) Correlation plot of ZNF536 and ADAMTS2. (**S**) Correlation plot of ZNF780B and ADAMTS2. (**T**) Correlation plot of ZNF844 and ADAMTS2.

Matrix ID	Name	Score	Relative score	Sequence ID	Start	End	Strand	Predicted sequence
MA0147.3	MA0147.3.MYC	7.3894053	0.8221168540792541	NC_000005.10:c179347461-179345461	1139	1150	+	GCCCAGGTGTCC
MA0147.3	MA0147.3.MYC	6.296079	0.7992913376472816	NC_000005.10:c179347461-179345461	607	618	+	TTCCATGTGACA
MA0147.3	MA0147.3.MYC	4.5303864	0.7624287325293405	NC_000005.10:c179347461-179345461	1919	1930	+	TCCCGCCTGCCT

Supplementary Figure 5. Three possible binding sites between MYC and the promoter of ADAMTS2.

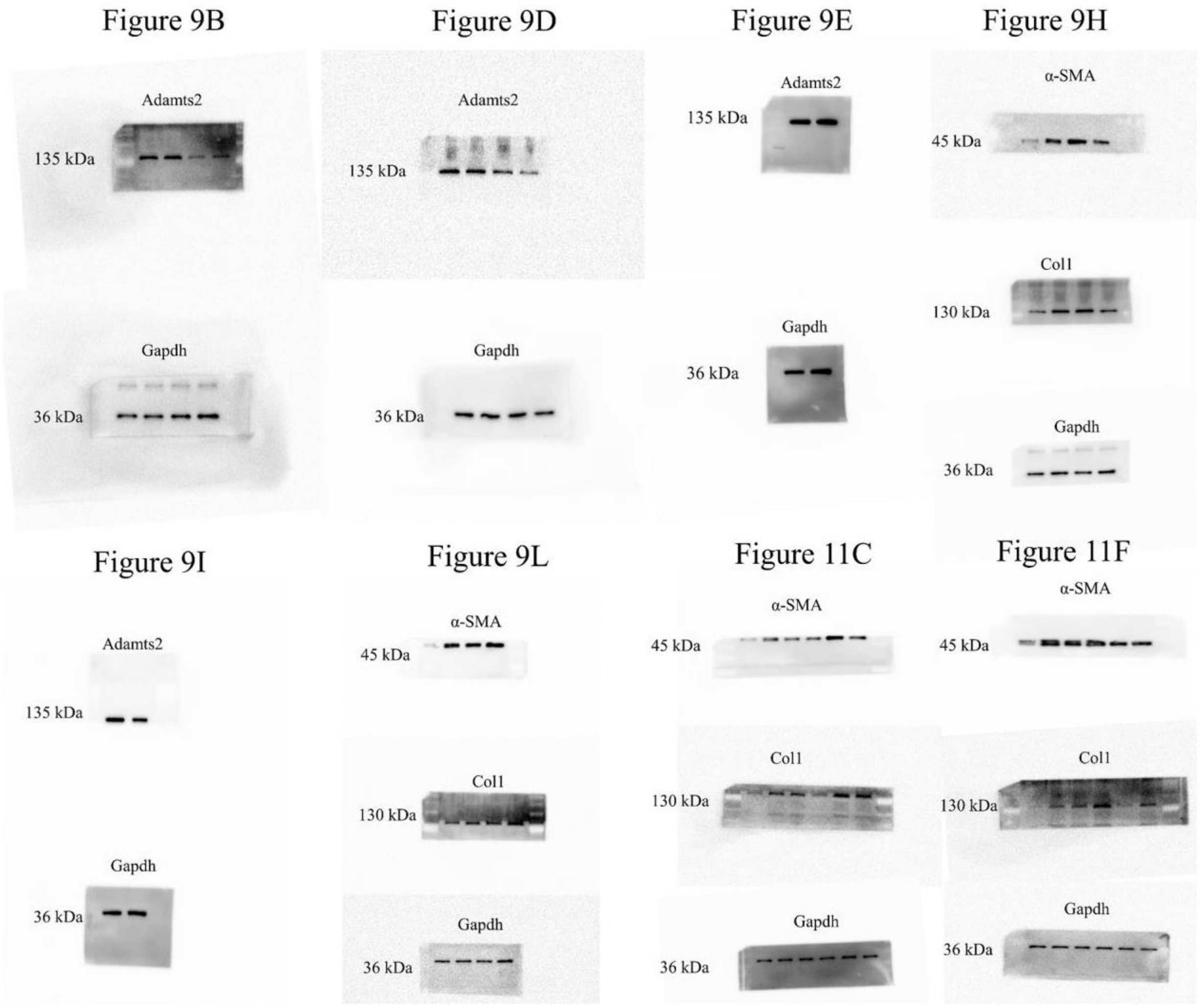

Supplementary Figure 6. Uncut membranes for western blot.
