# Supplementary material for: Identification of TGF-β-related genes in cardiac hypertrophy and heart failure based on single cell RNA sequencing
Source: Aging (Albany NY). 2023 Jul 26;15(14):7187–218. doi: 10.18632/aging.204901 (PMC10415570; doi:10.18632/aging.204901)
Supplement: Supplementary Tables 2 and 3 [file aging-15-204901-s003.pdf]

Supplementary Table 2. Cell markers used for cell annotation.

Gene	Cell_type
CD79A	B cells
CD79B	B cells
IGHD	B cells
IGHM	B cells
MS4A1	B cells
ACTC1	Cardiomyocytes
COX6A2	Cardiomyocytes
CRYAB	Cardiomyocytes
DES	Cardiomyocytes
MYBPC3	Cardiomyocytes
MYH7	Cardiomyocytes
MYH7B	Cardiomyocytes
MYL3	Cardiomyocytes
PLN	Cardiomyocytes
TNNI3	Cardiomyocytes
TNNT2	Cardiomyocytes
TPM1	Cardiomyocytes
AQP1	Endocardial cells
CLU	Endocardial cells
CYP1B1	Endocardial cells
EMCN	Endocardial cells
NPR3	Endocardial cells
RAMP2	Endocardial cells
VWF	Endocardial cells
AQP1	Endothelial cells
CLDN5	Endothelial cells
FABP4	Endothelial cells
FABP5	Endothelial cells
PECAM1	Endothelial cells
RAMP2	Endothelial cells
RGCC	Endothelial cells
VWF	Endothelial cells
C1R	Fibroblasts
COL1A1	Fibroblasts
COL6A3	Fibroblasts
DCN	Fibroblasts
EFEMP1	Fibroblasts
FBLN1	Fibroblasts
FBN1	Fibroblasts
IGF1	Fibroblasts
LUM	Fibroblasts
MGST1	Fibroblasts
SCN7A	Fibroblasts
SERPINF1	Fibroblasts
AIF1	Myeloid cells
C1QB	Myeloid cells
C1QC	Myeloid cells
CXCL8	Myeloid cells
HLA-DRB1	Myeloid cells
IL1B	Myeloid cells

ITGAX	Myeloid cells
LYZ	Myeloid cells
S100A9	Myeloid cells
SCN7A	Neuron
CD247	NK
CLIC3	NK
FCGR3A	NK
GNLY	NK
KLRB1	NK
KLRF1	NK
PRF1	NK
PTPRC	NK
SPON2	NK
TRDC	NK
ABCC9	Pericytes
CALD1	Pericytes
CD36	Pericytes
CPE	Pericytes
IGFBP7	Pericytes
KCNJ8	Pericytes
LHFP	Pericytes
NOTCH3	Pericytes
PDGFRB	Pericytes
RGS5	Pericytes
STEAP4	Pericytes
ACTA2	Smooth muscle cells
CALD1	Smooth muscle cells
IGFBP5	Smooth muscle cells
IGFBP7	Smooth muscle cells
LHFP	Smooth muscle cells
MYH11	Smooth muscle cells
PDGFRB	Smooth muscle cells
TAGLN	Smooth muscle cells
TAGLN	Smooth muscle cells
TPM1	Smooth muscle cells
TPM2	Smooth muscle cells
CD3D	T cells
CD3E	T cells
CD3G	T cells
IL7R	T cells
PTPRC	T cells
TRAC	T cells
TRBC2	T cells

Supplementary Table 3. Top five DEGs for cell clusters.

Gene	p_val	avg_log2FC	pct.1	pct.2	p_val_adj	Cluster
DCN	0	4.86033225383683	0.943	0.14	0	0
ACSM3	0	4.84943856649452	0.653	0.036	0	0
NEGR1	0	4.3807661059158	0.774	0.035	0	0
LUM	0	4.17344564299228	0.494	0.042	0	0
CDH19	0	3.92780518891926	0.737	0.018	0	0
RYR2	0	6.41775782329685	0.991	0.11	0	1
TTN	0	5.68238251857937	0.998	0.195	0	1
FGF12	0	4.96914952486419	0.93	0.054	0	1
CTNNA3	0	4.95286939204842	0.96	0.055	0	1
DMD	0	4.85986399702598	0.947	0.182	0	1
GZMK	0	3.12519579885936	0.424	0.019	0	2
IL7R	0	3.01077025286076	0.5	0.02	0	2
CD69	0	2.94537206115857	0.726	0.106	0	2
CXCR4	0	2.9188029665928	0.886	0.227	0	2
TRAC	0	2.59700805711885	0.653	0.058	0	2
HLA-DRA	0	4.24285489822362	0.849	0.253	0	3
CXCL8	0	4.18398707519192	0.562	0.053	0	3
C1QA	0	4.06026658748762	0.71	0.028	0	3
CD74	0	3.78416950025651	0.887	0.354	0	3
C1QC	0	3.77207331588078	0.638	0.012	0	3
GNLY	0	4.2851508393933	0.882	0.075	0	4
NKG7	0	3.87804636348164	0.984	0.138	0	4
GZMB	0	3.83851658763401	0.925	0.071	0	4
PRF1	0	3.71707226409034	0.89	0.065	0	4
FGFBP2	0	3.20214523026835	0.737	0.039	0	4
RGS5	0	5.28421822246012	0.911	0.058	0	5
ACTA2	0	4.51987305658022	0.81	0.067	0	5
TAGLN	0	4.1472989179207	0.689	0.123	0	5
NDUFA4L2	0	4.09931917981521	0.779	0.056	0	5
AGT	0	3.6876745069365	0.74	0.041	0	5
SLC9A3R2	0	4.04008473381861	0.851	0.079	0	6
IFI27	0	3.79608410198314	0.825	0.121	0	6
ID1	0	3.62468014382417	0.789	0.072	0	6
FABP4	0	3.4888404087723	0.79	0.123	0	6
VWF	0	3.34798840815497	0.773	0.08	0	6
S100A9	0	5.00310628777903	0.694	0.027	0	7
S100A8	0	4.94881242685836	0.571	0.013	0	7
LYZ	0	4.26141716783104	0.838	0.099	0	7
FCN1	0	3.70339245195514	0.765	0.014	0	7
LST1	0	3.50614744551876	0.881	0.085	0	7
ACKR1	0	3.97257247101983	0.741	0.007	0	8
STC1	0	3.4008436313059	0.46	0.023	0	8
CCL14	0	3.33867439895825	0.622	0.005	0	8
CLU	0	3.09401426633057	0.764	0.09	0	8
PLAT	0	2.82678356305652	0.702	0.027	0	8
TNNC1	1.43699903629166E-223	4.76107759314724	0.975	0.287	4.8743007311013E-219	9
MB	4.21235193032254E-220	4.51647790429309	0.967	0.282	1.4288297747654E-215	9
ACTA1	1.82481166635604E-210	4.85585977396702	0.979	0.314	6.18976117227969E-206	9
TNNI3	1.4818891019274E-208	4.70464102242041	1	0.347	5.02656783373773E-204	9
MYL2	3.9506659465879E-173	5.58323668400163	0.987	0.459	1.34006588908262E-168	9
CCL21	0	5.13205328081029	0.449	0.009	0	10
PKHD1L1	0	3.87291638172088	0.532	0.003	0	10
TFF3	0	3.28289030271198	0.427	0.016	0	10
EDN1	0	3.07489369875103	0.363	0.021	0	10

CLU	1.82380791808203E-267	3.32236229036408	0.701	0.108	6.18635645813424E-263	10
GPC6	5.22267657793858E-94	2.90893797803817	0.497	0.079	1.77153189523677E-89	11
POSTN	1.46559009070073E-43	3.06775284892027	0.27	0.046	4.97128158765689E-39	11
COL1A1	1.05211410073902E-41	3.67854698849994	0.417	0.11	3.56877102970677E-37	11
PDE3B	2.5572673369461E-17	3.44251152886124	0.362	0.167	8.67425080692116E-13	11
ACACB	6.3507832673874E-09	3.92448378530564	0.331	0.209	0.000215418568429781	11
IGKC	1.55078454812611E-217	7.58008961455903	0.734	0.141	5.26026118724375E-213	12
IGHG1	6.90440955885165E-207	6.42000621953637	0.404	0.041	2.34197572236248E-202	12
IGLC2	1.64680518815126E-172	7.27737071491919	0.511	0.077	5.58596319820907E-168	12
IGLC3	9.96566261184208E-162	6.70036041790492	0.369	0.042	3.38035275793683E-157	12
IGHA1	5.19386773046055E-86	5.83511690446958	0.376	0.073	1.76175993417222E-81	12
TPSAB1	0	6.85256327327856	0.635	0.005	0	13
TPSB2	0	6.36443747718339	0.806	0.01	0	13
SLC24A3	0	4.78566818689652	0.553	0.018	0	13
IL18R1	1.32075565447894E-289	4.58241578915418	0.594	0.042	4.48000317999258E-285	13
NTM	1.12275115714754E-252	4.77701416949464	0.494	0.033	3.80837192504445E-248	13
FGF12	1.45499579881097E-69	2.86759966933442	0.924	0.209	4.93534574956681E-65	14
FHL2	2.68435369638122E-65	3.37113657159157	0.924	0.25	9.10532773812509E-61	14
PPP1R12B	3.45017111772225E-56	2.86400244775912	0.911	0.279	1.17029804313139E-51	14
TMEM178B	1.09356460693364E-44	2.71873310457517	0.608	0.129	3.70937114671889E-40	14
ATP2A2	1.34309389359206E-43	2.71588179524891	0.797	0.261	4.55577448706428E-39	14
NRXN1	0	6.30799621129182	0.916	0.016	0	15
XKR4	0	5.25312021491104	0.773	0.013	0	15
KIRREL3	0	4.15004602677765	0.597	0.015	0	15
CADM2	1.83155279084511E-288	4.52409425928216	0.723	0.045	6.21262706654662E-284	15
NRXN3	1.72174344674826E-166	4.55304740506912	0.739	0.084	5.84015377137011E-162	15
