# Supplementary material for: Identification of TGF-β-related genes in cardiac hypertrophy and heart failure based on single cell RNA sequencing
Source: Aging (Albany NY). 2023 Jul 26;15(14):7187–218. doi: 10.18632/aging.204901 (PMC10415570; doi:10.18632/aging.204901)
Supplement: Supplementary Tables 7 and 8 [file aging-15-204901-s007.pdf]

Supplementary Table 7. Results of three machine learning algorithms.

Lasso	SVM-RFE	RandomForest	Intersection
ITGB5	FLNC	FLNC	FLNC
ENC1	NDRG4	NDRG4	CCL2
EGR1	CCL2	TGFB2	TANC2
ADAMTS2	TGFB2	CCL2	ADAMTS2
COL1A2	TANC2	TANC2	MIDN
SRPX2	ADAMTS2	MIDN	PRRC1
CCL2	ACACB	ADAMTS2	LDLR
CCL4	MIDN	ACACB	SOCS3
MRC2	PRRC1	LDLR	DYNLL1
SOCS3	LDLR	DYNLL1	RND3
CLDN5	SOCS3	PRRC1	NOTCH2
ADAMTS1	DYNLL1	NOTCH2	MRC2
PDLIM7	RND3	DLG1	DLG1
DYNLL1	NOTCH2	DOCK7	DOCK7
RND3	MRC2	SOCS3	EGR1
CCL3	DLG1	RND3	OTUD1
TULP4	DOCK7	MRC2	
NOTCH2	EGR1	MAMDC2	
PFKL	MAMDC2	EGR1	
FLNC	PLCG2	OTUD1	
LDLR	COPA		
GMDS	OTUD1		
AMOTL1	ZFP36		
PTPRE	TSC22D2		
PRRC1	CDC37L1		
MAP2			
SPON1			
COPA			
TANC2			
DEPTOR			
CSRP3			
OTUD1			
DOCK7			
PLCG2			
ROR1			
OTULIN			
MIDN			
IRF1			
PRELID2			
TNFRSF12A			
DLG1			
SRPK2			
MAP3K3			
TSC22D2			

Supplementary Table 8. Primers used for RT-qPCR.

Tanc2-Mouse	Forward primer: ACAAGCAGGGTCGTACTCC<br>Reverse primer: ACAAGCAGGGTCGTACTCC
Adamts2-Mouse	Forward primer: ACGCCTTTTCTACAACCTCAC<br>Reverse primer: GCCAGCCCATCACAGTTACT
Dynll1-Mouse	Forward primer: ATTGCGGCCCATATCAAGAAG<br>Reverse primer: GTGCCACATAACTACCGAAGTTT
Mrc2-Mouse	Forward primer: TCTCCCGGAACCGACTCTTC<br>Reverse primer: GGTCGAGCACATAGGTCTTCT
EGR1-Mouse	Forward primer: TCGGCTCCTTTCCTCACTCA<br>Reverse primer: CTCATAGGGTTGTTCGCTCGG
Otud1-Mouse	Forward primer: AGAGGCAGGACAAGTACCTGA<br>Reverse primer: CCCGTACACAGTCTTGCTGAC
Gapdh-Mouse	Forward primer: AGGTCGGTGTGAACGGATTTG<br>Reverse primer: TGTAGACCATGTAGTTGAGGTCA
Adamts2-Rat	Forward primer: TTGACGACAACAATGTCCTGGAA<br>Reverse primer: GGCGGCAGCCATACTTAGTGA
