# Supplementary material for: Identification of TGF-β-related genes in cardiac hypertrophy and heart failure based on single cell RNA sequencing
Source: Aging (Albany NY). 2023 Jul 26;15(14):7187–218. doi: 10.18632/aging.204901 (PMC10415570; doi:10.18632/aging.204901)
Supplement: Supplementary Tables 10 and 11 [file aging-15-204901-s009.pdf]

Supplementary Table 10. DETFs from GSE57338.

MEOX2
CSRNP3
CEBPD
BACH2
ZNF780B
PRDM1
CEBPB
ZNF610
RXRG
STAT3
BCL6
CSDC2
HOPX
HIF3A
EGR1
TEAD2
FOSL2
STAT4
ZNF676
IRX6
MYBL1
MEIS1
PRDM5
MYC
ZNF536
ZNF844
FOXP2
KLF15
ARNTL
ZNF483
CREB5
AFF3
SOX6
TEAD4
GTF2IRD1
NME2

Supplementary Table 11. Correlation between DETFs and hub genes.

TF	Gene	Cor	Pvalue	Regulation
AFF3	MRC2	0.442788806576612	1.84337004855542E-16	positive
CREB5	MRC2	0.480273793822713	1.82117672807951E-19	positive
CREB5	OTUD1	0.424601307932975	3.94100506772248E-15	positive
CREB5	TANC2	0.481197897939709	1.51880204113577E-19	positive
CSDC2	MRC2	-0.412047710164256	2.93761537234927E-14	negative
CSDC2	TANC2	-0.486776754754533	5.01614442809115E-20	negative
EGR1	OTUD1	0.442261092529332	2.01993211272779E-16	positive
HIF3A	MRC2	-0.418972581936567	9.80094370176029E-15	negative
HIF3A	TANC2	-0.408220138963945	5.33104346125116E-14	negative
MEIS1	MRC2	-0.404653939452163	9.22559922506808E-14	negative
MYC	ADAMTS2	0.465141370499743	3.29738532962804E-18	positive
MYC	EGR1	0.43630434024952	5.61025684118326E-16	positive
NME2	ADAMTS2	0.518968863540368	5.5611329003376E-23	positive
PRDM1	EGR1	0.410437253435248	3.77829471021524E-14	positive
PRDM1	TANC2	0.441181915584052	2.43421243531372E-16	positive
SOX6	ADAMTS2	-0.426011755224098	3.12823900404172E-15	negative
TEAD2	TANC2	-0.416329429166315	1.49460768407431E-14	negative
ZNF483	ADAMTS2	-0.429603452122813	1.72879640458759E-15	negative
ZNF536	ADAMTS2	-0.406651564364507	6.79087417949834E-14	negative
ZNF780B	ADAMTS2	-0.563580888780271	1.23615362726687E-27	negative
ZNF844	ADAMTS2	-0.483993837640592	8.73923511224908E-20	negative
